# The amygdalo-motor pathways and the control of facial expressions

**DOI:** 10.3389/fnins.2014.00043

**Published:** 2014-03-19

**Authors:** Katalin M. Gothard

**Affiliations:** Department of Physiology, The University of ArizonaTucson, AZ, USA

**Keywords:** *Macaca mulatta*, social behavior, neurophysiology, cingulate cortex, emotion, neuroanatomy, facial nucleus, interoception

## Abstract

Facial expressions reflect decisions about the perceived meaning of social stimuli and the expected socio-emotional outcome of responding (or not) with a reciprocating expression. The decision to produce a facial expression emerges from the joint activity of a network of structures that include the amygdala and multiple, interconnected cortical and subcortical motor areas. Reciprocal transformations between these sensory and motor signals give rise to distinct brain states that promote, or impede the production of facial expressions. The muscles of the upper and lower face are controlled by anatomically distinct motor areas. Facial expressions engage to a different extent the lower and upper face and thus require distinct patterns of neural activity distributed across multiple facial motor areas in ventrolateral frontal cortex, the supplementary motor area, and two areas in the midcingulate cortex. The distributed nature of the decision manifests in the joint activation of multiple motor areas that initiate the production of facial expression. Concomitantly multiple areas, including the amygdala, monitor ongoing overt behaviors (the expression itself) and the covert, autonomic responses that accompany emotional expressions. As the production of facial expressions is brought into the framework of formal decision making, an important challenge will be to incorporate autonomic and visceral states into decisions that govern the receiving-emitting cycle of social signals.

Both human and non-human primates use facial expressions to communicate their emotions and intentions. As a motor act, a facial expression is the reflection of a decision. In a strictly social context, facial expressions are produced either *to initiate* a social exchange, or *to respond* to others. The decision to produce one facial expression in lieu of another (or none at all) depends on the emotional state of the agent, the sensory-motor state of the agent's face, and the evaluation of the ongoing social situation (e.g., what expression had been emitted, who emitted it, the agent's relationship with the emitter, who else was present, and what the expected social gains and losses associated with possible responses are).

Traditionally, the circuit that controls facial expressions is conceptualized as a sequence of transformations that begins with perceiving the expressions of others, proceeds to extracting the socio-emotional significance of the perceived signals, and is completed by choosing and executing a motor response. This conceptualization suffers from several shortcomings. It implies unidirectionality, ignoring the role of feedback and the possibility that the status of the face and of the autonomic nervous system can directly influence the decision. It also implies that the decision can be confined to a structure located between the perceptual and the motor segments of this sequence. Implicit in this theory is the assumption that there should exist a neural signature of the decision at one central point within the circuit.

Alternatively, communication with facial expressions may occur as a single or multiple closed processing loops that carry out parallel reciprocal transformations between sensory and motor processes. These processes are informed by visceral inputs, and the predicted socio-emotional value of the available choices. This alternative suggests that the decision to produce an expression does not take place at an anatomically distinct decision node; rather it emerges from the activity of the entire circuit.

Recent experimental findings support this alternative. Neurons in both the primate amygdala and midcingulate cortex respond during the perception *and* production of facial expression (Livneh et al., 2012), suggesting that the neural signature of the decision process could be captured by monitoring neutral activity in these (or other) motor or limbic areas. Obtaining these data is limited only by our ability to record simultaneously the activity of ensembles of neurons from multiple brain areas. As this technology is emerging, it is worth contemplating where we should place the recording probes to best understand the circuits that support the receiving-emitting cycle of facial expressions? The sensory-perceptual aspects of social decision making have received ample attention in the literature, while the motor aspects have been less often addressed. The remainder of this article will highlight the anatomical aspects of the motor circuit involved in the production of facial expression that designate these areas as potential targets for future neurophysiological scrutiny.

Theoretically, a network involved in decisions about the use of facial expressions is be expected to contain: (1) last order motor neurons that directly innervate the facial muscles, (2) a network of motor cortical neurons that innervate the last-order motor neurons, (3) neurons that signal the emotional state of the agent, (4) somatosensory-proprioceptive neurons that signal the current state of the agent's face, and (5) neurons that signal the motivation, or social “justification,” to make a facial expression. With the exception of the motor neurons located in the facial nucleus (Jenny and Saper, 1987; Welt and Abbs, 1990) the other four types of neurons are located in multiple areas. For example, sensory-motor representations of the face are found in the parietal cortex (Avillac et al., 2005), the insula (Schneider et al., 1993), and in motor and premotor cortical areas (Gentilucci et al., 1988; Graziano et al., 1994). Information about the faces of others is also distributed; face identity and emotional expressions are processed concurrently in the amygdala (Nakamura et al., 1992; Gothard et al., 2007), the insula (Phillips et al., 1997), and in multiple face patches of the temporal and frontal cortex (Hasselmo et al., 1989; Tsao et al., 2006, 2008; Romanski, 2012).

## The motor control of facial expressions

Facial movements can be (1) voluntary, coordinated by cortical pathways, (2) reflexive, or (3) driven by central pattern generators coordinated by subcortical motor pathways, located mainly in the brainstem. Species-specific defensive behaviors and vocalizations, are typically orchestrated by specialized cell clusters in the periaqueductal gray (Jürgens and Ploog, 1970; Bandler and Shipley, 1994). Likewise the hypothalamus coordinates action patterns that are part of more complex ritualized behaviors such as courtship and mating, that may include facial displays (MacLean, 1990). These subcortical areas are hardly sufficient, however, to voluntarily direct a facial expression toward an individual of interest, as it happens during non-ritualized social interactions. Subcortical areas might be fast and efficient to extract general information, such as danger signals (Pessoa and Adolphs, 2010), but do not have the neural machinery to extract from faces subtle signals that inform our moment-to-moment decisions during social interactions (e.g., mock or heartfelt expressions of fear or happiness). Association areas in temporal and prefrontal cortices process the details of facial expressions and face-voice combinations to interpret their significance in the ongoing socio-emotional context. The output of these areas is critical for selecting choices of reciprocation and for estimating the outcome of each choice. The decision is ultimately reflected in the activity of motor areas that control directly the voluntary movements of the face.

Compared to the voluntary control of the limbs, the voluntary control of the face is poorly understood. While limbs execute movements such as reaching and grasping with kinematics that can be precisely measured, the muscles of facial expressions rearrange the configuration of the facial features to express emotions. Emotions are more difficult to quantify than arm kinematics, but even if this obstacle could be overcome, facial expressions can be produced even in the absence of emotion. The dissociation between the voluntary and emotional production of facial expressions has been amply documented in stroke patients with damage to different motor areas. Patients with strokes in the territory of the middle cerebral artery (primary motor and premotor areas) cannot produce a symmetrical, voluntary smile, nevertheless can smile normally in response to jokes (Monrad-Krohn, 1924; Hopf et al., 1992; Dawson et al., 1994; Töpper et al., 1995; Trepel et al., 1996). These findings suggest the existence of an alternative “limbic” pathway that controls facial expressions. Indeed, patients with strokes in the territory of the anterior cerebral artery, affecting the midcingulate area, are able to make voluntary facial movements but are unable to produce spontaneous emotional expressions (amimia) (Wilson, 1924; Feiling, 1927; Karnosh, 1945).

**Figure 1:**
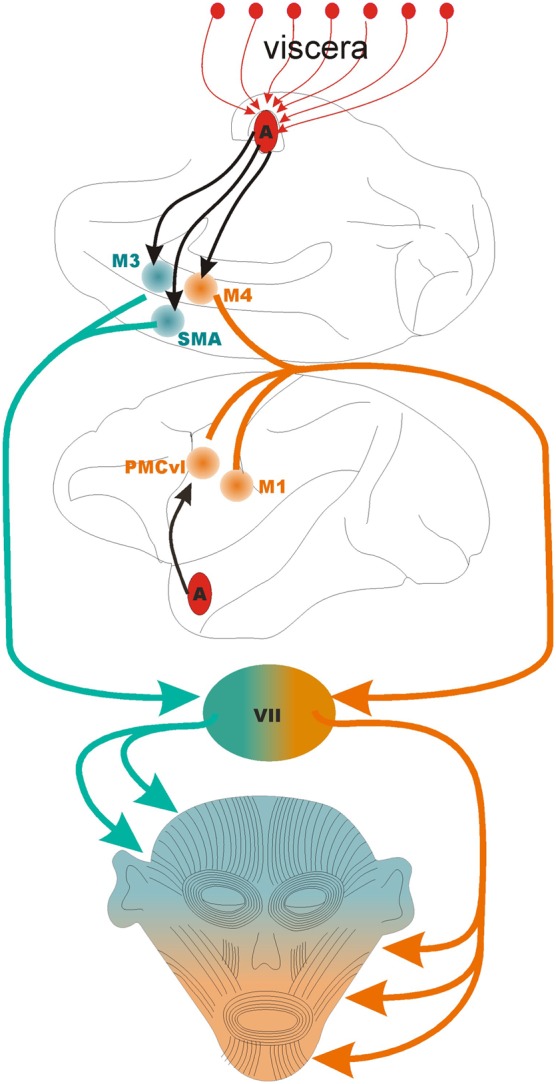
**The motor control of the face**. The lower half of the face is controlled by the coordinated activity of three motor areas: M1, primary motor cortex; PMCvl, premotor cortex ventrolateral division; and M4, caudal face area of the midcingulate cortex. The upper half of the face is controlled by the coordinated activity of two motor areas: SMA, supplementary motor area; and M3, the anterior face area of the midcingulate cortex. The black arrows indicate direct projections from the basal nucleus of the amygdala to PMCvl, M3, M4, and SMA, The first segment of the orange and green lines indicate the corticobulbar tract. VII, pontine facial nucleus that contain the motor neurons that synapse on the muscles of facial expressions. The medial division of the facial nucleus contains the motor neurons that control muscles in that upper half of the face (in green) while the lateral division contains the neurons that control the muscles in the lower part of the face (in orange). Note that the amygdala receives multiple lines of viscerosensory input (red arrows, top) that are likely integrated in the output directed at facial motor areas.

The cortical motor areas involved in production of facial expressions include: the primary motor cortex, the ventrolateral premotor cortex, the supplementary motor area, and two motor areas of the dorsal midcingulate (Morecraft et al., 2001, 2007). The localization of the two face areas in the midcingulate cortex is based on the work of Vogt (2009), who identified in the cingulate cortex a subgenual, an anterior (rostral to the genu of the corpus callosum), and a supracallosal portion (dorsal to the corpus callosum). The supracallosal region has been designated the midcingulate. The midcingulate has been further divided in anterior and posterior midcingulate, which contains two premotor areas for the face: a rostral area in the anterior portion of the midcingulate, designated by Morecraft et al. (2004) as M3, and a caudal area, at the border between the anterior and posterior divisions of the midcingulate, designated as M4 by the same authors (Figure 1).

The face area of the *primary motor cortex* innervates motor neurons in the lateral segment of the contralateral facial nucleus that control the lower facial muscles (Morecraft et al., 2001). The primary motor cortex also controls the muscles involved in mastication and other jaw movements that are innervated by trigeminal motor fibers.

The face area in the *ventrolateral regions of the premotor cortex (PMCvl*) directly innervates motor neurons in the lateral segment of the contralateral facial nucleus that control the lower facial muscles (Morecraft et al., 2001). In general, the premotor cortex initiates movements triggered by external cues (Murata et al., 1997; Fogassi et al., 2001; Mushiake et al., 2006). For facial expressions the external cues might be the facial expressions of others arriving to the PMCvl from temporal cortices and the amygdala (Avendaño et al., 1983). Notably, the PMCvl area is critical for linking the perception and production of actions, a process thought to be instantiated by mirror neurons (Di Pellegrino et al., 1992; Gallese et al., 1996). A full mirror neuron system for facial expression, akin to the mirror neurons for limb movements, has not been experimentally confirmed. However, suggestive findings indicate that in monkeys, neurons in the ventral premotor cortex respond during the observation and execution of a particular form of facial expression (Ferrari et al., 2003).

The *supplementary motor cortex (SMA)* directly innervates motor neurons in the medial segment of the facial nucleus (medulla) that control the upper facial muscles (Morecraft et al., 2001). Compared to the PMCvl that controls movements triggered by external cues, the SMA appears to control self–initiated movements (Eccles, 1982; Romo and Schultz, 1987; Lang et al., 1994). If this division of labor holds for facial expressions, the SMA might coordinate self-initiated expressions that involve the upper facial musculature (e.g., winking, scowling).

The *anterior and caudal face areas of the midcingulate cortex* (Picard and Strick, 2001), designated as M3 and M4 by Morecraft et al. (2001) show further specializations. M3 gives rise to projections that target bilaterally the medial segments of the facial nucleus harboring the motor neurons that supply the upper facial muscles and the muscles that move the ears (in monkeys) (Figure 1). Projections originating from M3 also target the reticular formation of the brainstem that contains autonomic centers likely to become activated during emotional states (Porrino and Goldman-Rakic, 1982). M3 is in position, therefore, to coordinate both the overt (behavioral) and covert (autonomic) expression of emotions. The caudal motor area, M4, (located at the border between the anterior and posterior midcingualte) targets the lateral regions of the facial nucleus, especially the motor neurons that supply the upper lip (Morecraft et al., 2007). In theory, damage to M4 should impair elevation of the contralateral upper lip, a movement involved in appeasing gestures in monkeys, in smiling in humans, and in disgust in both species. Indeed, in humans, surgical resection of the medial wall of the hemisphere that includes M4 impairs smiling. The deficits caused by M4 damage is absent during voluntary smiles (Hopf et al., 1992) which stands in contrast to the lower facial weakness caused by damage to primary motor cortex or to the PMCvl. It appears, therefore, that M4 is mostly involved in the emotional control of facial expressions. This area also appears to respond to the expected reward of actions, such as looking at certain visual targets (McCoy and Platt, 2005). This is not surprising in light of the massive convergent input from reward-related and motor areas of the brain (Vogt and Pandya, 1987; Morecraft and Van Hoesen, 1998). Perhaps the most eloquent example of the critical role that the dorsal cingulate cortex plays in the decision to socially interact with others is the dramatic reduction of movement and speech in a condition known as akinetic mutism (Cairns et al., 1941). The “cingulate syndrome,” a variant of akinetic mutism includes as additional symptoms flat affect, reduced alertness, and autonomic abnormalities (Cummings, 1993). When patients recover, they report intact memory for the numerous requests to respond to questions and commands and explain their lack of responses by a complete lack of desire to interact with others. The cingulate syndrome is significant because it highlights the cingulate as the site where the limbic system gains access to the motor system (Morecraft et al., 2007). Indeed multiple information processing streams converge in the cingulate cortex: multisensory temporal and frontal areas (Baleydier and Mauguiere, 1980), pain pathways (Hutchison et al., 1999; Koyama et al., 2001; Eisenberger et al., 2003; Botvinick et al., 2005; Iwata et al., 2005), and reward pathways (Amiez et al., 2005; Chang et al., 2013). Several aspects of affect (Critchley et al., 2003), cognitive control (Davis et al., 2005; Rudebeck et al., 2006; Hayden and Platt, 2007; Womelsdorf et al., 2010), and motor control (West and Larson, 1995; Russo et al., 2002) have been attributed to the cingulate cortex (reviewed by Shackman et al., 2011).

## A role of the amygdala in the production of facial expressions

By virtue of its vast connectivity to visual association areas in the temporal and frontal cortices (Amaral et al., 1992), the primate amygdala is specialized to evaluate facial expressions. While the amygdala might not be necessary for the motor elaboration of facial expressions, it appears critical for selecting the expressions that are most appropriate for a given social context. Monkeys and humans with bilateral lesions of the amygdala appear less reserved when encountering strangers and produce more affiliative displays (Meunier et al., 1999; Emery et al., 2001; Adolphs, 2010; Bliss-Moreau et al., 2013). In light of these findings, it is not surprising that electrical stimulation of the amygdala, and seizures originating therein, cause facial movements in both humans and monkeys (Baldwin et al., 1954; Feindel and Penfield, 1954; Feindel, 1961; van Buren, 1961; Gloor, 1975; Bossi et al., 1984; Hausser-Hauw and Bancaud, 1987; Fish et al., 1993). The output of the amygdala might influence the choice of facial expressions because it signals the identity, facial expression, and gaze direction of others (Leonard et al., 1985; Gothard et al., 2007; Hoffman et al., 2007; Gamer and Büchel, 2009) or the subjective impression elicited by face stimuli (Wang et al., 2013). During naturalistic social interactions a class of specialized cells become active in the amygdala that respond when monkeys fixate their gaze on the eyes of other monkeys. A subset of these “eye cells” respond only during eye contact (Zimmerman et al., 2012) which enhances the emotional impact of facial expressions. The eye cells are unconventional in that their activity depends on the dynamic exchange of gaze between the viewer and the individual the viewer interacts with. Such interplay between gaze perception and the decision to make (or not) eye-contact is analogous to the reciprocity of the social signals mediated by the cingulate cortex (Amodio and Frith, 2006). Indeed, the duration of eye contact is a strong predictor of facial-expression reciprocation in monkeys (Mosher et al., 2011) and in humans (Usui et al., 2013).

Anatomically, the amygdala forms a closed processing loop with both the anterior cingulate cortex and with area M3 (the anterior component of the midcingulate) (Morecraft et al., 2007). M3 projects to the basal and accessory basal nuclei of the amygdala and the basal nucleus of the amygdala gives rise to feedback projections to all subdivisions of the cingulate cortex (Amaral et al., 1992; Morecraft et al., 2007). The massive interconnectivity between the amygdala and the cingulate cortex might explain the similarity of cellular responses in these two areas. Neurons in the amygdala and in the midcingulate face areas respond to the production of facial expressions by monitoring the expressions of self. Activity in these areas becomes more synchronous during the execution of facial expressions, with neural activity in the amygdala leading neural changes in the midcingulate cortex. In both areas, however, the activity of individual cells may precede or follow the productions of facial expressions (Livneh et al., 2012). Fine-grain analysis of the temporal relationship between the firing rate changes and the onset of muscular activity (measured with intramuscular electromyography) have demonstrated that, at least in the amygdala, neurons respond primarily *after* the onset of facial activity (Fuglevand et al., 2012). As such, the amygdala might be responding primarily to the sensory consequences associated with the production of facial expressions. This finding, together with the role of the amygdala in monitoring the facial expressions of others (Gothard et al., 2007) suggest a mirror neuron system for facial expressions of self and of others (Dapretto et al., 2006).

## Emotion-to-motor transformation in the amygdalo-cingulate circuits

Functional predictions based on the anatomical connectivity of the amygdala and the cingulate cortex, are gradually reinforced by neural data and from clinical observations. Patients with motor conversion syndromes (DSM V, American Psychiatric Association, 2013) are either paralyzed or produce abnormal movements in the absence of damage to motor pathways. These patients appear to have a hyperactive amygdalae manifested in increased anxiety, increased galvanic skin response and baseline cortisol, heightened vigilance, and decreased vagal tone (Voon et al., 2010). Select case studies indicate that in these patients the activity levels in the amygdala and the motor areas are inversely related (Kanaan et al., 2007; Voon et al., 2010).

Further evidence for emotion-to-motor transformation comes from new research on the putative role of visceral-somatic loops in social behavior. Since 1872, when Darwin related facial expressions to emotions and implicitly to internal states the brain circuits involved and their connectivity became better known. It has been proposed that decision making is strongly influenced by bodily states (Damasio, 1996; Critchley and Harrison, 2013) and these signals arise in the visceral afferents. The midcingulate and the amygdala receive signals from the viscera via the nucleus of the solitary tract and parabrachial nuclei (Amaral et al., 1992; Craig, 2002; Khalsa et al., 2009) and via the insula which integrates interoceptive and exteroceptive signals, also projects to the amygdala and the midcingulate (Mufson et al., 1981; Vogt and Pandya, 1987; Craig, 2002). Interoceptive afferents, therefore, may modulate both the perception and the production of facial expressions. Indeed, neurons in the amygdala and cingulate cortex discharge in phase with the cardiac and respiratory cycle (Frysinger and Harper, 1986, 1989) and in response to stimulation of the vagal nerve (e.g., Bachman et al., 1977; Hassert et al., 2004; Conway et al., 2006). An astonishing anatomical observation about the vagus nerve highlights the role of visceral inputs for decision making: even though the descending axons in the vagus control the majority of internal organs, 80% of the fibers are ascending, carrying signals from the viscera to the brain (Sengupta and Shaker, 2005). Given the oscillatory nature of visceral afferents (e.g., systole/diastole), it is unsurprising that the perception of cutaneous stimuli and emotional facial expressions has been shown to depend on the phase of the cardiac cycle (Gray et al., 2009, 2012).

While the ascending segment of the visceral-limbic and visceral-cortical loops may influence decisions (Craig, 2002; Prinz, 2004), descending segments trigger autonomic changes during the production of emotional expressions. A functional overlap in these loops might explain the concomitant visceral and facial-motor effects cause by electrical stimulation in the amygdala and the cingulate (Pool and Ransohoff, 1949; Baldwin et al., 1954; van Buren, 1961; Jürgens and Ploog, 1970).

In summary, recent progress in our understanding of the neural mechanisms involved in the perception and production of facial expressions is sufficient to bring facial expressions into the theoretical framework of decision making. Several elements of current decision-making theories, such as prior distributions, probabilities, loss and gain functions, are applicable to social transactions via facial expressions. Social decision-making has already been tested empirically and analyzed using the formalisms developed by neuroeconomics (Sanfey et al., 2003; Hayden et al., 2007; Frith and Singer, 2008; Lee, 2008). The next major challenge will be to include facial expressions in these formalisms and the visceral states that contribute to the decision process.

### Conflict of interest statement

The author declares that the research was conducted in the absence of any commercial or financial relationships that could be construed as a potential conflict of interest.
